# Supplementary material for: Systematic Profiling of Ale Yeast Protein Dynamics across Fermentation and Repitching
Source: bioRxiv. 2023 Sep 22:2023.09.21.558736. Preprint. [Version 1] doi: 10.1101/2023.09.21.558736 (PMC10543003; doi:10.1101/2023.09.21.558736)
Supplement: Supplementary file 1 [file NIHPP2023.09.21.558736v1-supplement-2.pdf]

bioRxiv preprint doi: https://doi.org/10.1101/2023.09.21.558736; this version posted September 22, 2023. The copyright holder for this preprint (which was not certified by peer review) is the author/funder, who has granted bioRxiv a license to display the preprint in perpetuity. It is made available under aCC-BY 4.0 International license.

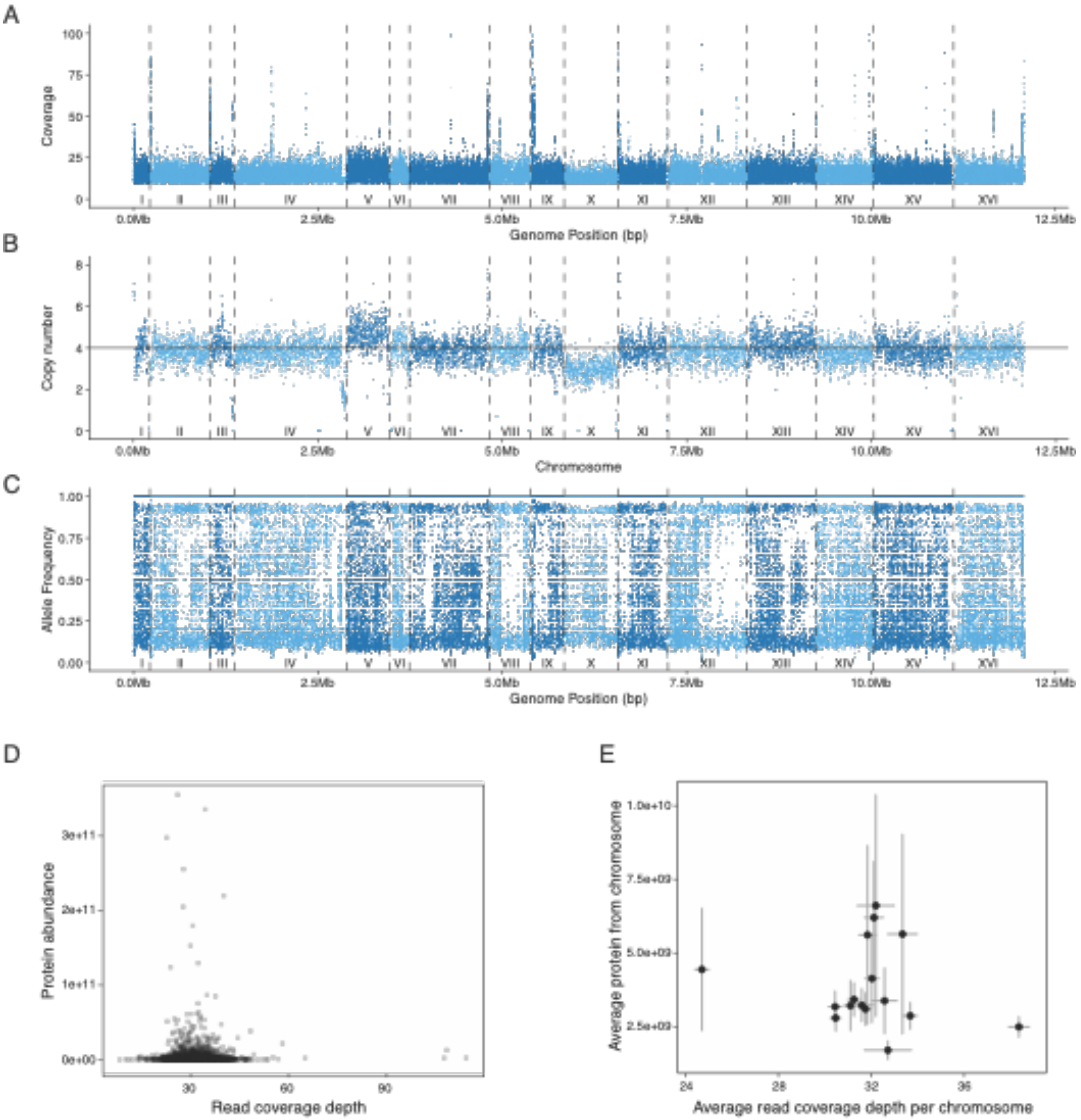

Supplementary Figure 1. Genomic characteristics of Wyeast 3068. A) Mean sequencing coverage across the Wyeast 3068 genome. Each dot represents the mean coverage (y-axis) of a particular locus along the genome position (x-axis). B) Local copy number as an average read depth over 1000bp windows normalized to the known ploidy of 4n. C) Allele frequencies (y-axis) of called variants. (Note that genome positions in A, B, and C have been concatenated for whole-genome display). D) Comparison between read depth (x-axis) at each gene and level of the corresponding protein abundance (y-axis). Adjusted R^2=0.00008246, p≤0.2703. E) Comparison

bioRxiv preprint doi: https://doi.org/10.1101/2023.09.21.558736; this version posted September 22, 2023. The copyright holder for this preprint (which was not certified by peer review) is the author/funder, who has granted bioRxiv a license to display the preprint in perpetuity. It is made available under aCC-BY 4.0 International license.

between average read depth/chromosome (x-axis) and average LFQ abundance of corresponding protein by chromosome (y-axis), adjusted R^2=-0.04194, p≤0.5392.

bioRxiv preprint doi: https://doi.org/10.1101/2023.09.21.558736; this version posted September 22, 2023. The copyright holder for this preprint (which was not certified by peer review) is the author/funder, who has granted bioRxiv a license to display the preprint in perpetuity. It is made available under aCC-BY 4.0 International license.

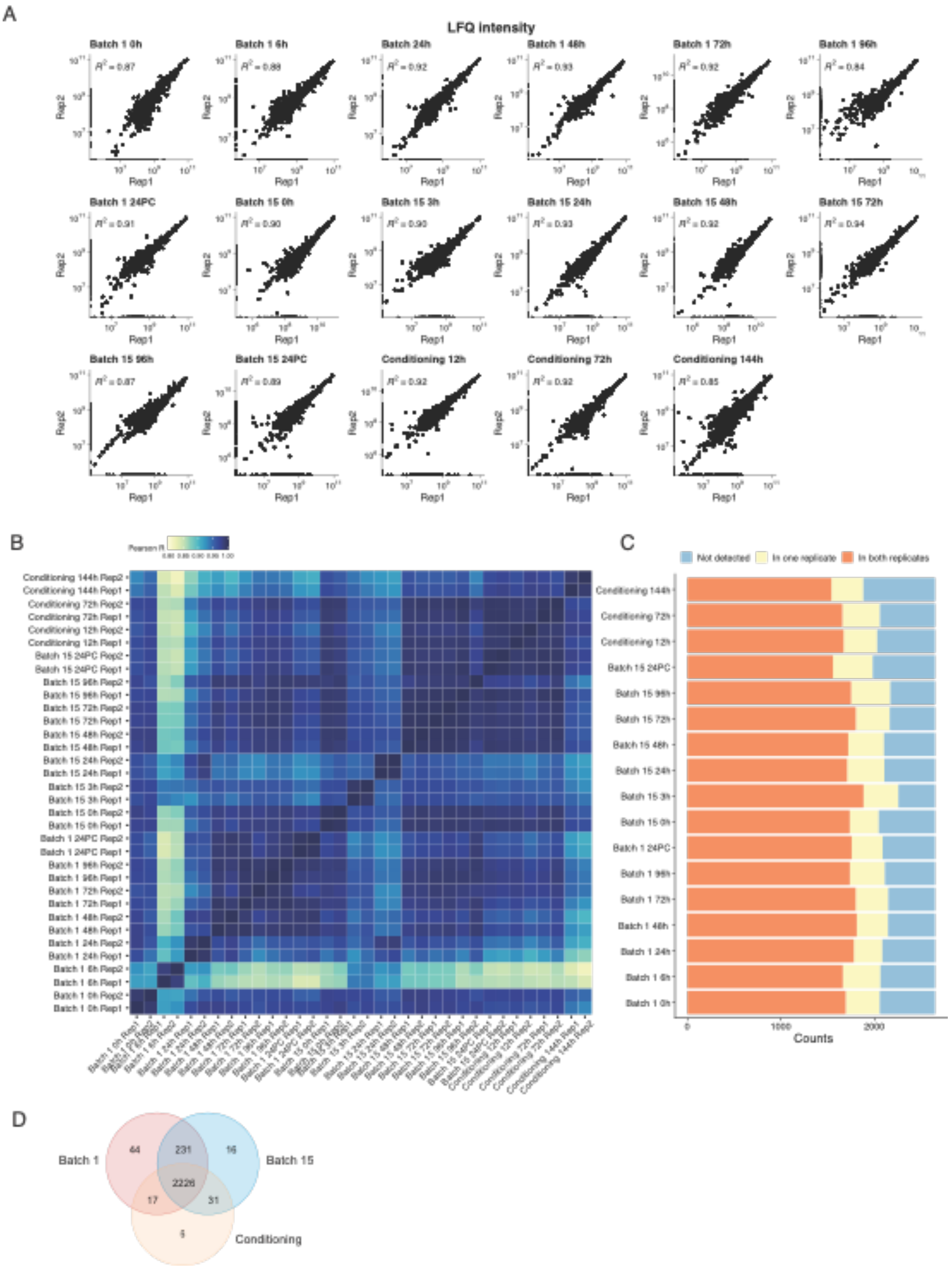

bioRxiv preprint doi: https://doi.org/10.1101/2023.09.21.558736; this version posted September 22, 2023. The copyright holder for this preprint (which was not certified by peer review) is the author/funder, who has granted bioRxiv a license to display the preprint in perpetuity. It is made available under aCC-BY 4.0 International license.

Supplementary Figure 2. Fermentation proteomics summary statistics. A) Scatterplots depicting the correlation between replicates 1 and 2 for each fermentation time point assayed. Axes indicate the log-transformed observed LFQ intensity calculated using MaxQuant. B) Pairwise similarity matrix showing the extent of correlation across all sampled replicates and time points in the dataset. C) Barplots summarizing the number of proteins detected in each time point colored by detection in both (orange), one (yellow), or undetected (blue). D) Venn diagrams summarizing the overlap of proteins detected across Batch 1 (red), Batch 15 (blue), and the Conditioning tank (orange).

bioRxiv preprint doi: https://doi.org/10.1101/2023.09.21.558736; this version posted September 22, 2023. The copyright holder for this preprint (which was not certified by peer review) is the author/funder, who has granted bioRxiv a license to display the preprint in perpetuity. It is made available under aCC-BY 4.0 International license.

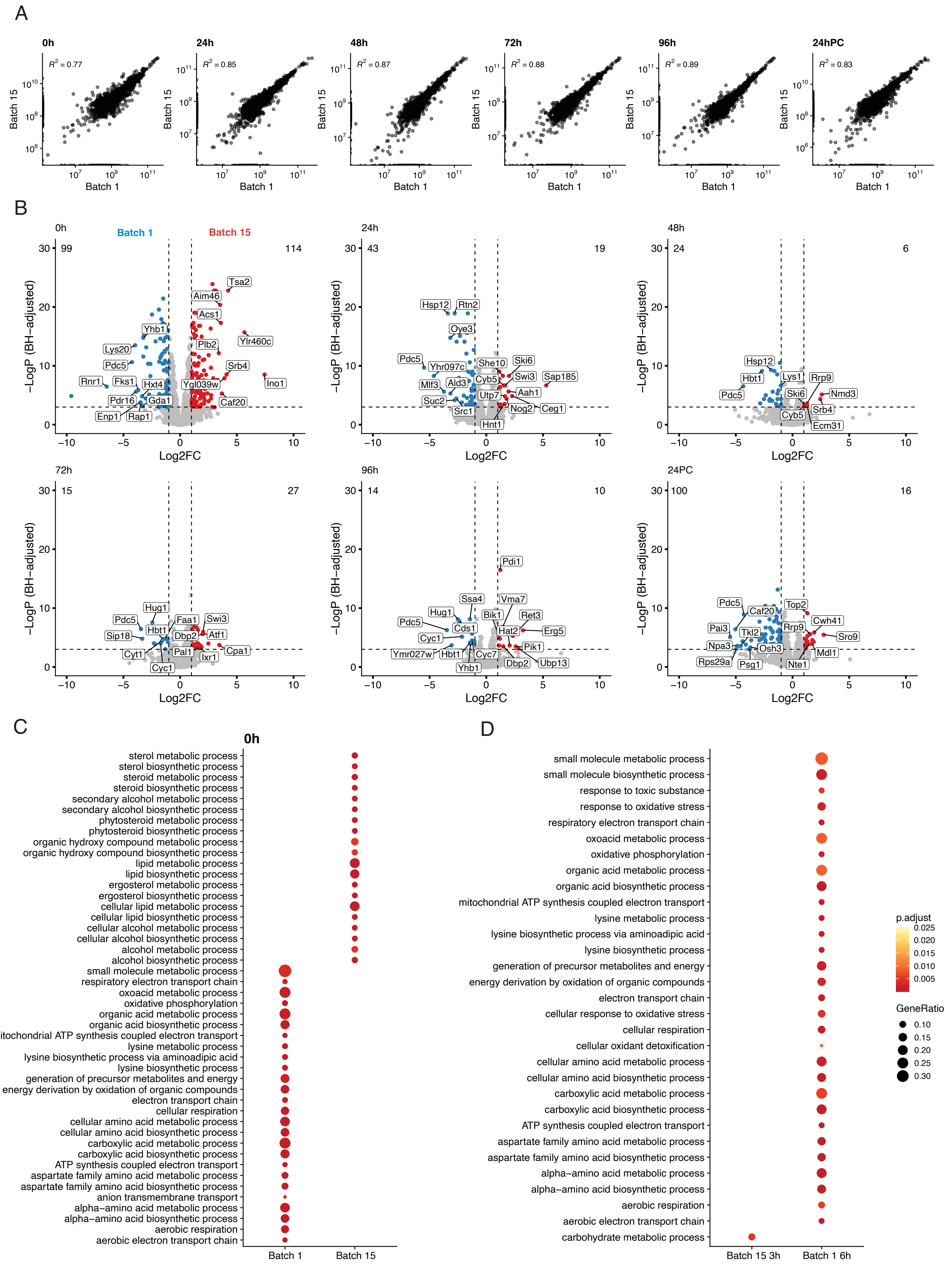

bioRxiv preprint doi: https://doi.org/10.1101/2023.09.21.558736; this version posted September 22, 2023. The copyright holder for this preprint (which was not certified by peer review) is the author/funder, who has granted bioRxiv a license to display the preprint in perpetuity. It is made available under aCC-BY 4.0 International license.

Supplementary Figure 3. Comparing differentially expressed proteins across shared time points between batches 1 and 15. A) Scatter plots of summed LFQ intensity across replicates comparing time points sampled in both Batch 1 (x-axis) and Batch 15 (y-axis) brewing cycles. B) Differentially expressed proteins across matched time points (0h, 24h, 48h, 72h, 96h, and 24PC) between Batches 1 and 15 depicted using volcano plots with $\log_2$ fold change (x-axis) and Benjamini-Hochberg adjusted p-value (y-axis). C) Dotplots with the top enriched GO terms for the matched starting time point across Batch 1 and Batch 15. D) Dotplots with top enriched GO terms across Batch 15 3h and Batch 1 6h time points. GO term analysis was performed on the biological function terms with a 5% FDR threshold and filtering terms to an adjusted p-value (Benjamini-Hochberg correction) of <0.05. Sizes of dots correspond to the ratio genes detected to the total genes annotated for a particular GO term.

bioRxiv preprint doi: https://doi.org/10.1101/2023.09.21.558736; this version posted September 22, 2023. The copyright holder for this preprint (which was not certified by peer review) is the author/funder, who has granted bioRxiv a license to display the preprint in perpetuity. It is made available under aCC-BY 4.0 International license.

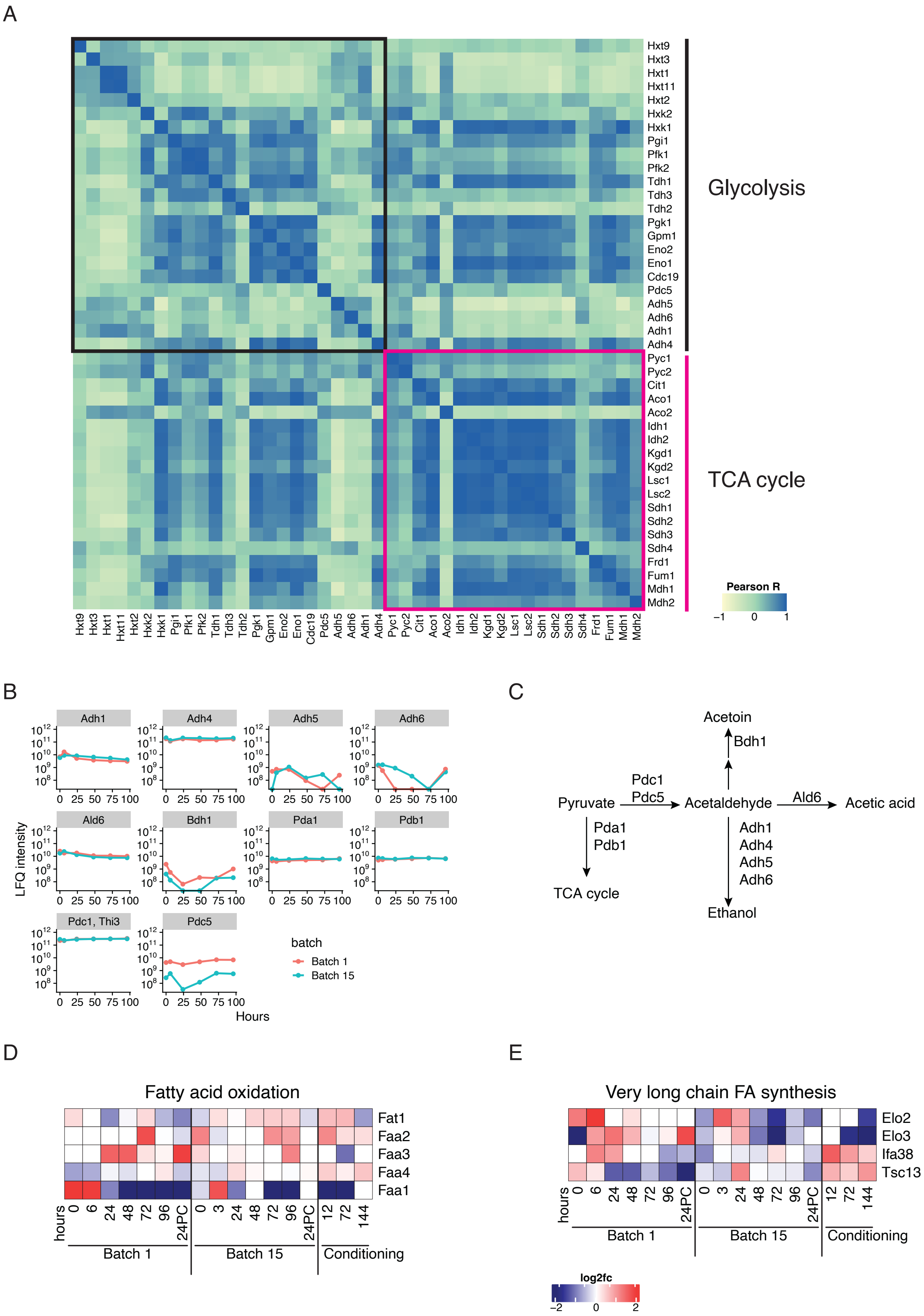

bioRxiv preprint doi: https://doi.org/10.1101/2023.09.21.558736; this version posted September 22, 2023. The copyright holder for this preprint (which was not certified by peer review) is the author/funder, who has granted bioRxiv a license to display the preprint in perpetuity. It is made available under aCC-BY 4.0 International license.

Supplementary Figure 4. Cataloging abundance changes in metabolic pathways. A) Pairwise Pearson correlation between proteins involved in the glycolysis and tricarboxylic acid (TCA) cycle pathways. B) LFQ protein abundance (y-axis) as a function of time (x-axis) for enzymes involved in pyruvate metabolism. Time points colored by batches 1 (red) and 15 (blue) C) Steps in pyruvate metabolism in yeast. D) Changes in fatty acid oxidation and E) very long chain fatty acid synthesis enzyme abundances, over both batches and during final conditioning, as $\log_2$ of row mean normalized abundance.

bioRxiv preprint doi: https://doi.org/10.1101/2023.09.21.558736; this version posted September 22, 2023. The copyright holder for this preprint (which was not certified by peer review) is the author/funder, who has granted bioRxiv a license to display the preprint in perpetuity. It is made available under aCC-BY 4.0 International license.

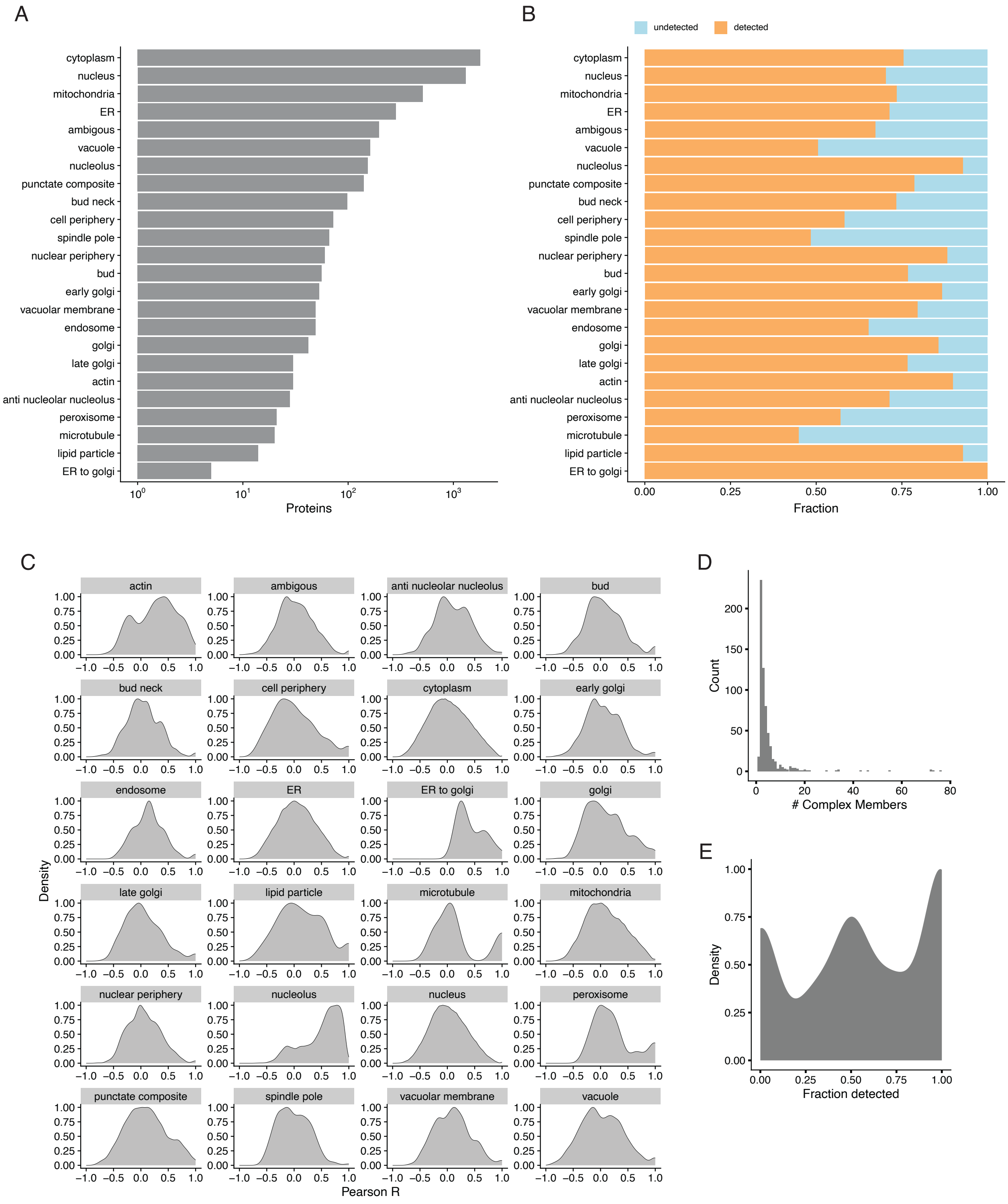

Supplementary Figure 5. Subcellular proteome analysis. A) Barplots representing the numbers of yeast proteins (x-axis) annotated by subcellular/organellar location (y-axis). Data curated from the Yeast GFP Fusion Localization database[40] and B) Fraction of proteins in each subcellular/organellar location detected in at least one time point across the brewing time course.

bioRxiv preprint doi: https://doi.org/10.1101/2023.09.21.558736; this version posted September 22, 2023. The copyright holder for this preprint (which was not certified by peer review) is the author/funder, who has granted bioRxiv a license to display the preprint in perpetuity. It is made available under aCC-BY 4.0 International license.

C) Densities of all Pearson correlation values calculated across all pairs of proteins across each annotated subcellular location and organelles. D) Histogram of the distribution of yeast protein complex sizes. Data obtained from Yeast complexome. E) Density plot showing the fraction of yeast protein complexes detected in the fermentation time course. Protein complex data curated from the EBI Complexome database[41].
